# P4G2Go: A Privacy-Preserving Scheme for Roaming Energy Consumers of the Smart Grid-to-Go

**DOI:** 10.3390/s21082686

**Published:** 2021-04-11

**Authors:** Aristeidis Farao, Eleni Veroni, Christoforos Ntantogian, Christos Xenakis

**Affiliations:** 1 Department of Digital Systems, University of Piraeus, 18534 Piraeus, Greece; arisfarao@unipi.gr (A.F.); veroni@unipi.gr (E.V.); 2 Department of Informatics, Ionian University, 49100 Corfu, Greece; dadoyan@ionio.gr

**Keywords:** anonymous credentials, Idemix, FIDO2, privacy-preserving, smart grid, anonymity

## Abstract

Due to its flexibility in terms of charging and billing, the smart grid is an enabler of many innovative energy consumption scenarios. One such example is when a landlord rents their property for a specific period to tenants. Then the electricity bill could be redirected from the landlord’s utility to the tenant’s utility. This novel scenario of the smart grid ecosystem, defined in this paper as Grid-to-Go (G2Go), promotes a green economy and can drive rent reductions. However, it also creates critical privacy issues, since utilities may be able to track the tenant’s activities. This paper presents P4G2Go, a novel privacy-preserving scheme that provides strong security and privacy assertions for roaming consumers against honest but curious entities of the smart grid. At the heart of P4G2Go lies the Idemix cryptographic protocol suite, which utilizes anonymous credentials and provides unlinkability of the consumer activities. Our scheme is complemented by the MASKER protocol, used to protect the consumption readings, and the FIDO2 protocol for strong and passwordless authentication. We have implemented the main components of P4G2Go, to quantitatively assess its performance. Finally, we reason about its security and privacy properties, proving that P4G2Go achieves to fulfill the relevant objectives.

## 1. Introduction

The smart grid [1] is becoming the next-generation power grid supporting bi-directional power and communication flows between utility companies and energy consumers. It delivers electricity from utilities to consumers while reducing costs and increasing reliability and transparency. The smart grid enables better pricing policy and can increase the potential of energy markets due to its flexible model. A new energy market has recently emerged through the vehicle-to-grid (V2G) networks, promoting the use of renewable energy resources and the concept of green energy. V2G has lately gained a lot of attention from the research community, since electric vehicles are expected to play a key role in the forthcoming years in the global effort for transportation to become environmentally sustainable.

Due to its flexibility in terms of charging and billing, the smart grid is an enabler of many interesting new application scenarios of power consumption and usage. One such use case, which is also the motivation of this work, is the following one. Consider a scenario where landlords rent their properties to tenants for a specific period for business or leisure purposes (Airbnb is an example of an online marketplace for renting houses [2]). According to the current metering and billing system, landlords must pay for their tenants’ energy consumption. However, tenants seem to expect unrestricted consumption leading to excessive charges (e.g., charging of electric vehicles by tenants) [3,4,5]. In such scenarios, the adoption of smart grid effectively can solve this issue by billing the actual consumer instead of the landlord [6]. Smart meters can be programmed to charge the tenant through routing consumption measurements from the landlord’s utility to the tenant’s utility. In this paper, we propose Grid-to-Go (G2Go) where the aforementioned scenario can be realized. Nowadays, the G2Go concept seems to be more relevant than ever. Due to the unprecedented mobility restrictions enforced by the COVID-19 pandemic, the “work from home” model has gained significant traction, making professionals realize that they can provide their services from anywhere in the world. These professionals have now the opportunity to embrace the location-independent working lifestyle of digital nomads that allows them to travel and work remotely from anywhere in the Internet-connected world. A recent report reveals that the number of digital nomads in the United States has soared by nearly 50% since 2019 [7]. Even after the mobility restrictions are lifted, the new digital nomads are expected to retain their flexible workspaces [8,9], since many corporations are shifting towards permanent remote working.

Enabling the G2Go concept improves the efficiency and flexibility of the smart grid, but it also raises great privacy issues and challenges. As G2Go leverages the main architectural components of the location-fixed smart grid networks, it inherits the smart grid privacy considerations which are related to the fact that the smallest detail of household energy consumption can be revealed, including energy consumer habits or detection of the residents’ absence from the property [10,11]. On top of that, G2Go also shares most of the privacy-related concerns encountered in V2G, as both networks permit roaming and consumer mobility, a feature that could be exploited to track location patterns and disclose habits [12]. Therefore, the G2Go requires a new approach in order to cover the privacy and security requirements of smart grids and V2G networks simultaneously. This paper proposes P4G2Go, a privacy-preserving scheme designed to address the needs defined by the roaming consumer scenario. P4G2Go utilizes well established, secure cryptographic protocols and assembles them into a novel scheme that provides strong security and privacy assertions for roaming energy consumers against honest but curious utilities, as well as adversaries who may monitor the smart grid. More specifically, at the heart of P4G2Go lies the Idemix anonymous credential system that enables selective disclosure of attributes that prove that an energy consumer (i.e., the tenant) is legitimate without however disclosing their real identity to untrusted utilities. Idemix provides unlinkability of charging sessions and energy consumption by roaming consumers [13,14], regardless of the number of times the same credential has been used for verification. This is of paramount importance as colluding utilities can try to track the trajectory of roaming consumers as they move from one place to another. P4G2Go also integrates the Fast Identity Online 2 (FIDO2) [15] to achieve passwordless authentication and enable the mobile device that consumers habitually carry to be the secure container of the Idemix credentials. Finally, in order to establish a trustworthy environment in the smart grid ecosystem, P4G2Go incorporates the MASKER protocol (developed and evaluated in our previous paper [11]), which aims at providing a privacy-preserving data aggregation solution to protect the energy consumption readings from internal and external adversaries who may monitor the smart grid network. To assess the performance of P4G2Go, we have implemented the incorporated technologies including Idemix. Numerical results show that P4G2Go can efficiently operate a significant number of verification requests. Finally, we evaluate the security and privacy properties of P4G2Go to prove its effectiveness against a set of privacy breach attempts. In summary, the paper makes the following contributions:

- Define the G2Go concept and present its functional, security and privacy requirements. To the best of our knowledge, this is the first time a scenario for roaming energy consumers is being proposed by the literature.
- Propose P4G2Go, a privacy-preserving scheme designed for the G2Go concept based on well-established security and privacy-preserving technologies.
- Assess P4G2Go’s performance and qualitatively reason about its security and privacy properties. For this purpose, we have implemented the main components of P4G2Go including the Idemix anonymous credential system.

The paper unfolds as follows: Section 2 discusses the related work, while Section 3 presents the characteristics of the G2Go concept as well as its security and privacy requirements. Next, Section 4 investigates the technologies leveraged in P4G2Go and provides a high-level description of the architecture. Section 5 elaborates on P4G2Go operations describing in detail all the required steps. Section 6 includes the performance evaluation of our scheme, while Section 7 discusses its security and privacy properties. Finally, Section 8 concludes the paper.

## 2. Related Work

While this paper is the first work that defines the G2Go scenario for traveling consumers who occasionally reside in places other than their home, incorporating for this purpose technologies for anonymous authentication and billing to protect the consumer’s data security and privacy, such technologies have been previously combined in the context of electric mobility (e-Mobility) and vehicle-to-grid (V2G) networks. More specifically, there is a plethora of works aiming at addressing the many privacy-related challenges that networks which foresee consumer mobility (e.g., V2G) face. Electric vehicles (EVs) require frequent stops for charging, a procedure that starts with the authentication of both EV’s and owner’s identities and usually concludes with the billing process, raising numerous security and privacy concerns [16,17]. Since several similarities can be identified in the security and privacy concerns described for the roaming EV charging scenario and the proposed G2Go application scenario, we have considered previous research on privacy-preserving charging schemes for roaming EVs as related work for this paper, focusing on the technologies used and the level of privacy protection they offer. There is a vast literature concerned with finding solutions to the most prominent privacy-related problems in the V2G ecosystem. A large part of it is dedicated to proposing anonymous authentication and authorization mechanisms for EVs, considering also the identity of their users [18,19,20,21,22]. Another common problem that has received significant attention from researchers is the billing and payment processes [12,23,24,25,26,27,28]. Research works that deliver solutions to the aforementioned critical topics but do not specifically consider the roaming charging scenario, are out of the scope of this study.

In both V2G and G2Go, the challenge lies in building a robust and computationally efficient scheme following the privacy-by-design approach. More specifically, the proposed solutions should satisfy the security and privacy requirements, and at the same time allow critical information to reach the operators to be able to effectively monitor the grid and ensure the accountability and non-repudiation in the system. Up to this day, only few privacy-preserving charging schemes for roaming EVs have been published, each exhibiting one or more limitations according to the existing literature [16,29].

The first study that proposed a privacy-preserving protocol for e-Mobility charging was [25]. Höfer et al., after identifying by means of a Privacy Impact Assessment the privacy gap in the draft ISO/IEC 15118 standard which specifies the V2G communication interface for EV charging, designed and implemented its privacy-enhanced version called POPCORN. Similar to P4G2Go, POPCORN leverages anonymous credentials to enable selective disclosure of attributes using the Idemix cryptographic protocol suite, whereas it employs group signatures for EVs to sign the meter readings during charging for protection against cheating vehicles. The authors had to introduce additional actors in the ecosystem defined by the ISO/IEC 15118 standard for handling the payments between providers and resolving possible disputes. While some of its privacy properties have been formally verified, several shortcomings have also been identified, such as the fact that no strong unlinkability properties have been formally proven for the presented scheme [30].

Another privacy-preserving charging protocol for roaming EVs has been proposed in [26], considering the hosts’ renewable energy sources as potential electricity suppliers other than the grid. To this end, the authors introduced in their scheme a fair billing functionality, all the while maintaining the EV user’s identity and location privacy, as well as session unlinkability through the use of different pseudonyms. Moreover, designed back in 2014, the scheme foresees the utilization of the now outdated smart cards for users to store their sensitive data (i.e., cryptographic keys) for authentication purposes. According to [31] however, the roaming user’s privacy can yet be compromised, since the home and host suppliers have direct communication, disclosing both the home and visiting area of the consumer based on the location of the host supplier’s charging stations.

Saxena et al. [32] proposed a mutual authentication scheme based on a bilinear pairing technique to preserve the privacy of an EV’s information from different entities participating in the grid (e.g., aggregators), both in the home and the visiting V2G networks. While the scheme has shown through comprehensive security analysis to provide resistance against various attacks, it has been identified that it bears significant additional overhead due to the use of computationally inefficient cryptographic primitives [33,34,35]. A charging protocol extended to support payment transactions in line with the principles of the Secure Electronic Transaction (SET) protocol, was proposed in [27]. This work provides anonymous authorization and payment simultaneously through the use of dual signatures and pseudonym IDs, protecting user’s privacy from both home and host suppliers. To do so, apart from a certificate authority, a broker entity was also added in the system in order to act as a mediator between suppliers. Both the security and the efficiency of the proposed protocol were not verified by the authors.

Finally, the work carried out in [28] has revealed the shortcomings of existing and upcoming Plug-and-Charge standards (ISO 15188, Open Charge Point Protocol, and Open Interchange Protocol) where, based on the authors’ analysis, no measures have been defined for protecting the privacy-sensitive charging and billing user data, and avert the generation of movement profiles. The authors have in turn proposed extensions to the aforementioned protocols to address these flaws, leveraging group signatures and a Direct Anonymous Attestation technique that employs a Trusted Platform Module installed in the vehicle, introducing only minimal overhead to the original Plug-and-Charge process.

In summary, the related work on V2G networks copes with various privacy and security challenges, including charging session linkability, security attacks at the level of vehicle software/firmware, vehicle ID tracking, obtaining location related information and extracting driving preferences of users. However, the security and privacy requirements of G2Go extend well beyond the basic requirements of V2G. As G2Go is a hybrid concept, combining features from V2G and location-fixed smart grid networks, G2Go inherits also the security and privacy requirements of the latter, where determining personal behavior patterns and the use of specific appliances is possible, allowing the real time surveillance of the household by adversaries, who are in position to detect residents’ absence from the property and launch targeted home invasions (elderly, children), or having third parties use consumption data for profiling and marketing purposes. Therefore, a new privacy-preserving scheme is required that will fulfil not only the security and privacy requirements of V2G, but also of the traditional smart grid networks.

## 3. The G2Go Concept

### 3.1. Definition and Participants

**Figure 1:**
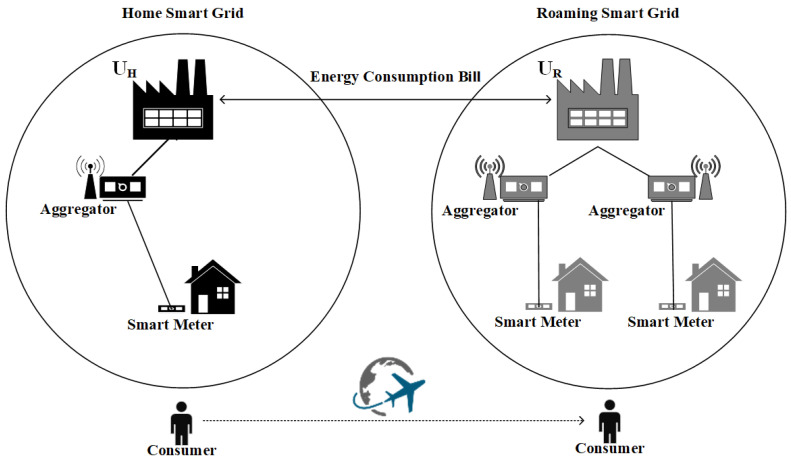
Relation between the P4G2Go entities.

The G2Go allows roaming consumers to have full control over their energy consumption and billing in every property they visit or rent, even in cross-border cases, as long as the related smart grid technology is supported. The basic scenario of G2Go unfolds as follows (see Figure 1). A roaming *Consumer* is a subscriber to their *Home Utility* company (denoted as *U_H_* hereafter). At some point, the *Consumer* travels and becomes a tenant for a specific period of time in a different place, which is served by a different utility company defined as *Roaming Utility* (denoted as *U_R_*). G2Go enables landlords to avoid being charged for the consumed energy by their tenants. Instead, the tenants will be charged for their exact consumption by their *U_H_*. Evidently, a service level agreement (SLA) between the *U_H_* and the *U_R_* should define the way the *U_R_* will be reimbursed for the energy consumption of the tenant. For example, small payments may be mutually discarded.

A notable advantage of G2Go is the disincentivization of tenants to needlessly consume energy when they reside in a rented property for a short period (such as in cases of short-term rental agreements through Airbnb). Thus, G2Go contributes towards building a wiser energy consumption mentality promoting environmental awareness. On the other hand, landlords’ profit is indirectly increased, since the energy consumption and the related bill is decoupled from the landlord’s. Therefore, the property owners can reduce the cost of renting their apartments (i.e., positive externalities), making them more affordable for tenants and thus, more attractive. One can draw parallels between the G2Go scenario and the roaming scenario in mobile networks. In the latter case, a mobile user wants to access the roaming network and get charged by their home operator. However, in contrast to G2Go, mobile operators have long established trust relationships between home and roaming networks. For the G2Go concept to be realized, in a similar manner to the mobile operators, roaming agreements must be put in place between energy suppliers to facilitate flexible charging for consumers traveling domestically or abroad [25].

The G2Go enables interesting business cases and new actors. In particular, we identify three primary stakeholders: (i) the end-users, (ii) the property owners (landlords), and (iii) the utility companies. A fourth stakeholder is the secondary market that may emerge, in order to address the need for new specialized software for renting, marketing and advertising, etc. On a technical level, the entities that participate in the G2Go scenario are defined as follows (see also Table 1): (i) The *Utilities* are responsible for supplying electric energy and billing the *Consumers* for their consumption. In G2Go, we distinguish two different types of Utilities: the *U_H_* that supplies the *Consumer*’s home with electricity and the *U_R_* that supplies the rented property, (ii) The smart meters are responsible for collecting energy consumption packets. Each property (e.g., apartment, workplace) is bound to one smart meter, (iii) The aggregator, which acts as an intermediate node between the *Utility* and the smart meter, collects the consumption packets sent by smart meters and calculates the consumed energy in each property, (iv) The *Consumer* who is a person who has an official contract with their *U_H_*, and, (v) The *Consumer’s Device*, which is a mobile device that *Consumers* habitually carry along (denoted as *D_C_*), such as a smartphone or a tablet. Note that the *D_C_* is not part of the G2Go architecture, but it plays a significant role in the architecture of P4G2Go as we will analyze in Section 4.

### 3.2. Security Model

Taking into account the aforementioned participating entities and their relations, we draw the respective security model for our solution, relying on the following assumptions:

(1) Smart meters convey consumption readings and they are trustful. However, a malicious software injected after the proper deployment of the smart meter may try to obtain the readings or convey false information to aggregators.
(2) Aggregators follow the honest-but-curious model, which is what most related works on privacy-preserving aggregation depend on. According to this model, aggregators securely send valid and accurate energy consumption data without discarding or tampering the transmitted messages, but they may try to deduce information from the received messages.
(3) *U_R_* also follows the honest-but-curious model in the sense that they properly execute the involved protocols, but they are curious and may try to read the data received from other nodes in order to gain information. We assume that different *U_R_* may collude and combine legitimately acquired information in order to link the activities of the *Consumer*.
(4) The *U_H_* follows strict protocol procedures and is trusted by both *U_R_* and the *Consumer*.

### 3.3. Security and Privacy Requirements

As previously mentioned, G2Go is a hybrid concept combining features from V2G and location-fixed smart grid networks and as such, it inherits the security and privacy requirements of both. Since the security requirements of the smart grid ecosystem have been well-established by the literature, lately the focus appears to be shifting towards the privacy-related conditions that must be met by every proposed solution [36]. For solutions designed to address the needs of roaming consumers who reside in temporary accommodation and wish to be billed fairly for the energy they consume, we define the following security and privacy requirements after considering the smart grid’s architectural components, users’ security and privacy demands and the related research.

#### 3.3.1. Security Requirements

Since the smart grid involves network operations inherited from both traditional IT and electricity systems, we redefine the following set of standard security requirements applied to the former category within the G2Go concept [17,37,38], with the addition of the physical protection requirement, which has been elicited based on the known weaknesses of the smart grid components to physical attacks [11,39].

(S1) *Data confidentiality*: Consumption data must be available only to the responsible *Utility* and the *Consumer*. No entities may collude to gain information in order to track a *Consumer*’s activity.
(S2) *Data integrity and authenticity*: All data exchanged between the participating entities should be protected against alteration and replication. Each entity should be in a position to verify the source of the data received.
(S3) *Non-repudiation*: No *Consumer* should be able to deny their actions.
(S4) *Authorization and access control*: Access to the roaming service is granted only to legitimate *Consumers* registered at the *Utilities* that participate in the scenario.
(S5) *Accountability*: A *Consumer* should be held accountable for their actions.
(S6) *Physical protection*: Smart grid components should incorporate protection mechanisms to prevent being tampered with by adversaries with physical access.

#### 3.3.2. Privacy Requirements

The privacy requirements set for G2Go are mainly mobility-related and for this purpose, previous work considering the roaming electric vehicle charging scenario has been used as a basis for their definition [16,26,28]. The only exception to the above is the privacy-preserving data aggregation requirement, an objective of great significance for the users to be able to maintain their privacy, that was usually encountered in fixed-location smart grid networks [11,40,41] until recently [42]:

(P1) *Identity privacy*: *Consumer*’s true identity should only be known to their *U_H_*. *U_R_* authenticates *Consumers* only by their pseudonyms, and it should not be possible for adversaries to identify a *Consumer* by monitoring the grid.
(P2) *Location privacy*: There should be no way for colluding *U_R_* entities to track the trajectory of *Consumers*.
(P3) *Unlinkability*: Guarantees that different charging sessions from the same *Consumer* cannot be linked to each other.
(P4) *Minimum data disclosure*: Guarantees that suppliers should access *Consumer’s* data limited to the minimum required to bill them.
(P5) *Privacy-preserving data aggregation*: Aggregation of consumption data should happen in a secure and privacy-preserving manner that protects *Consumer’s* individual consumptions from being disclosed or modified by unauthorized parties, and prohibits the linkage of a property with a specific energy usage. Also, the end result of the consumption data aggregation should be computed correctly in order to charge the *Consumer*.

## 4. P4G2Go Technologies and Architectural Overview

### 4.1. P4G2Go Technologies

Now we briefly present the technological pillars of the P4G2Go scheme that jointly provide a privacy-by-design solution for the G2Go. We have designed P4G2Go on the grounds of well-established technologies with proven security and privacy properties: (i) Idemix, (ii) Trusted Execution Environment (TEE) and (iii) MASKER and (iv) FIDO2.

#### 4.1.1. Idemix

Idemix [43] is an anonymous credential system for selective disclosure of attributes to minimize revealing personal data in digital communications. Moreover, it provides privacy-preserving features such as anonymity, the ability to transact without revealing the identity of the transactor, and unlinkability, the ability of a single subject to send multiple transactions without revealing that these were completed by the same subject. Idemix is the crux of our proposed scheme; it will allow roaming *Consumers* to hide their real identity from *U_R_*, to prevent leakage of their private information. Generally speaking, the involved participants in Idemix are the user, an issuer and a verifier. The Idemix protocol consists of two basic functionalities. The first is the credential issuance, where the user (acting as a receiver) obtains credentials by the issuer. This credential consists of a set of attribute values, as well as cryptographic information that allows the credential’s owner (i.e., the user) to create a proof of possession. Each credential is issued on a pseudonym of the user. The user can generate an arbitrary number of pseudonyms using a private key called Idemix master secret. These pseudonyms are unlinkable in the sense that an entity cannot tell whether two pseudonyms originated from the same master secret. Moreover, revealing a pseudonym does not provide any information about the master secret. The use of pseudonyms generated by a secret key is analogous to traditional public key cryptography, where a public key is the identity of the user (e.g., as in Bitcoin), but unlike public key cryptography, in Idemix the user can generate as many public keys (i.e., pseudonyms) as they want from their private key (i.e., master secret). The second functionality of Idemix is credential proving, where a user (acting as a prover) must prove the possession of certain attributes to a verifier without necessarily revealing the values contained within them using zero-knowledge proofs. When showing a credential, the user can choose which of the credential’s attributes shall be revealed and which will be hidden. The user also generates a pseudonym (different from the one used to issue the credential) that will be used as a user reference by the verifier. In this way, both issuers and verifiers identify users only by (different) pseudonyms which cannot be linked.

An extended functionality of Idemix is the cryptographic primitive called verifiable encryption. The latter allows an Idemix credential owner to prove that their credential contains a special attribute which is in essence an encrypted value using the public key of an entity (a trusted third party or the credential issuer itself). This can be very helpful for cases where, for example, a verifier allows access to a service only if the received credential includes the (encrypted) ID card of the user. Thus, although the verifier cannot decrypt the ID card, it can validate the fact that the encrypted value of the ID card is indeed present in the credential (hence the term verifiable encryption). If de-anonymization is required, the verifier will convey the encrypted ID card to the owner of the public key (a trusted third party or the issuer) in order to decrypt (using the related private key) and reveal the real identity of the user. In P4G2Go we take advantage of a verifiable encryption attribute, in order to de-anonymize the *Consumer* and charge them when needed as we analyze below.

#### 4.1.2. Trusted Execution Environment (TEE)

The Trusted Execution Environment (TEE) [44] can be considered a sandbox capable of executing applications (named Trusted Applications). The isolation of the normal operating system from the TEE entails a secure environment, where applications of the normal world including malicious software are out of reach of sensitive data either stored in TEE or utilized by trusted applications. ARM TrustZone is an implementation of a TEE, which has gained particular attention, because ARM processors are omnipresent in the mobile market. Originally, the ARM TrustZone was introduced only for the Cortex-A processors (found in mobile devices), but more recently it has been extended to Cortex-M processors specially designed for embedded platforms, such as smart meters. In P4G2Go, the *D_C_* that supports an ARM TrustZone will be utilized for storing the Idemix anonymous credentials and the Idemix master secret, while smart meters and aggregators will also utilize ARM TrustZone to enhance the security properties of the MASKER protocol (see Section 4.1.3).

#### 4.1.3. MASKER

A vital part of P4G2Go’s architecture is the MASKER [11] protocol, which provides a lightweight privacy-preserving aggregation of consumption data. In MASKER, each participating smart meter shares with the *Utility* a series of securely generated pseudorandom values called masks. These mask values are used to hide the smart meter readings without loss of accuracy. The obfuscation is achieved by simply adding the random mask values to the consumption data. This way, an intermediate aggregator receives from the smart meter only masked consumption readings and cannot obtain the real consumption values. The aggregator sums all the masked data and provides the *Utility* with an aggregated value (which is the masked total consumption). The *Utility* simply performs a subtraction of the used masks from the aggregated value received by the aggregators, resulting in the real total consumption of the relevant smart meter. In other words, MASKER provides an additive homomorphic solution in a scalable and efficient manner, suitable for low capability devices such as smart meters. Only the smart meter can read the real energy consumption values. In this way, MASKER protects the consumers’ privacy by concealing the energy consumption and withstands against adversaries who may attempt to monitor the consumers’ activities and habits. Furthermore, MASKER achieves an accurate consumption data mechanism leading to a correct and fair billing method.

At the level of smart meters and aggregators, the performed sensitive computations in MASKER are protected by utilizing a TEE, which stores data and executes crucial operations. In particular, MASKER utilizes a TEE in aggregators and smart meters for: (i) key generation and storage; (ii) performing secure computations (i.e., additions) for deriving the readings in a masked form. In essence, TEEs provide an extra layer of security, safeguarding smart meters and aggregators from malware that may attempt to tamper the randomness of the generated keys. The inner working of MASKER and its technicalities can be found in [11].

#### 4.1.4. Fast Identity Online 2 (FIDO2)

The FIDO2 protocol [15,45,46] enables users to leverage common devices such as smartphones (also known as FIDO2 devices) to provide a passwordless authentication [47] to services. First, the user must register their mobile device to a FIDO2 server, using authentication mechanisms supported by the device such as fingerprints (or any other biometric modality or authentication mechanism, such as a pin). The exact authentication mechanism can be imposed by the security policies of the FIDO2 server. At this point, the FIDO2 server attests the user’s device and then the latter, acting as a FIDO2 authenticator, generates a public/private key pair. The private key will be stored in the TEE of the device while the public key will be transferred to the FIDO2 server. After the registration of the device, the FIDO2 server can authenticate the user of that specific device. This is performed with public key cryptography using a challenge-response protocol. That is, the FIDO2 server sends a challenge to the device, and the latter requires the authentication of the user in order to release the private key. In case of successful authentication, the device signs the challenge and sends it back to FIDO2 server for verification. Evidently, the device of the user must be secure from attacks that could attempt to retrieve the private key.

FIDO2 includes several advantageous characteristics compared to standard authentication procedures. First, it provides strong authentication based on the use of biometric authentication while the overall user experience is frictionless since the user neither needs to type passwords in such small devices, nor has to remember passwords in the first place. In P4G2Go, FIDO2 is primarily used for the authentication of the *Consumer* with the *U_H_* (i.e., the Idemix issuer).

### 4.2. P4G2Go Architecture

**Figure 2:**
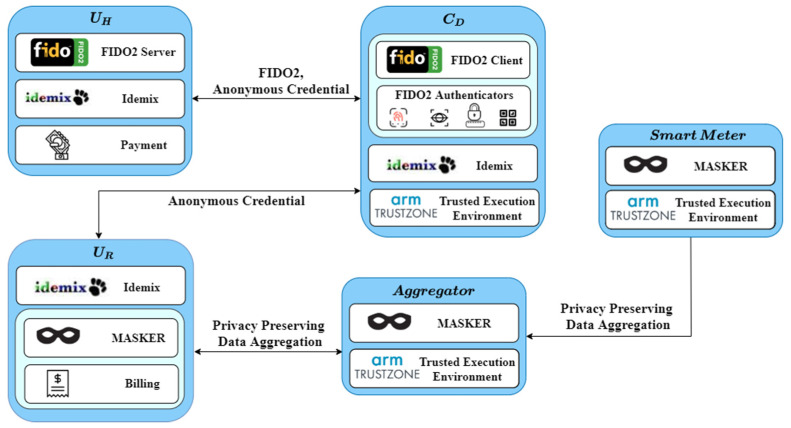
P4G2Go architectural components.

In this section, we present a blueprint of the P4G2Go architecture along with the protocol stack of each entity participating in G2Go as shown in Figure 2:

***D_C_*:** The mobile device of the user is the gist of our architecture. P4G2Go takes advantage of the FIDO2 protocol to utilize the mobile device of the user as a gateway for accessing the service offered by our solution. In particular, the *D_C_* allows users to request the issuance of cryptographic credentials from the *U_H_* and is responsible for revealing issued credentials to *U_R_*. The *D_C_* incorporates also a Trusted Execution Environment (TEE) to store Idemix credentials along with the Idemix master secret key. In this way, a *Consumer* can access and use their Idemix anonymous credentials using their mobile device eliminating the need for smart cards or other cumbersome solutions that would undermine the overall user experience.

***U_H_*:** This entity is an Idemix issuer allowing users to issue cryptographic credentials, from their verified identity attribute, directly to their mobile device and then use them to access *U_R_*. *U_H_* has an identity repository that stores its customers’ profiles and issued credentials. It also encapsulates a FIDO2 server for undertaking FIDO2 authentication. Finally, the *U_H_* will also receive the payment of the energy consumption bill from the *Consumer*.

***U_R_*:** This entity is an Idemix verifier. It will validate the received anonymous credentials of the *Consumer*, checking whether they are eligible to use the service or not. Moreover, the *U_R_* is responsible for unmasking the masked aggregated values to calculate the total consumption bill using the MASKER protocol.

**Smart meters and aggregators**: These two entities run the MASKER protocol for privacy-preserving aggregation of consumption data. Additionally, both entities include an ARM TrustZone TEE to further safeguard MASKER’s critical operations.

## 5. P4G2Go Operations

P4G2Go consists of the following individual operations: (i) The *Credential issuance* in which the *Consumer* is authenticated to their *U_H_* using FIDO2 and subsequently requests the issuance of credentials; (ii) *Credential verification and privacy-preserving data aggregation* where, as its name implies, the user shows their credentials to the *U_R_* and in case of a successful verification, the *Consumer* can start using electrical appliances or charge their devices. The MASKER protocol enables privacy-preserving data aggregation of consumption measurements which are conveyed to the *U_R_* for billing; (iii) Finally, the *Billing and payment* procedure which involves the de-anonymization of the *Consumer* by the *U_H_* in order to provide the electricity bill to *Consumer*. To perform the P4G2Go operations, we assume that the *D_C_* has installed a mobile application that implements the required functionality of P4G2Go. We also assume that the smart meter includes a local interface (such as a touch/display screen) that allows user interaction with smart meter functionalities (e.g., showing energy usage). The work in [48] analyzes several value-added services specifically based on the local interface of such smart meters.

### 5.1. Credential Issuance

**Figure 3:**
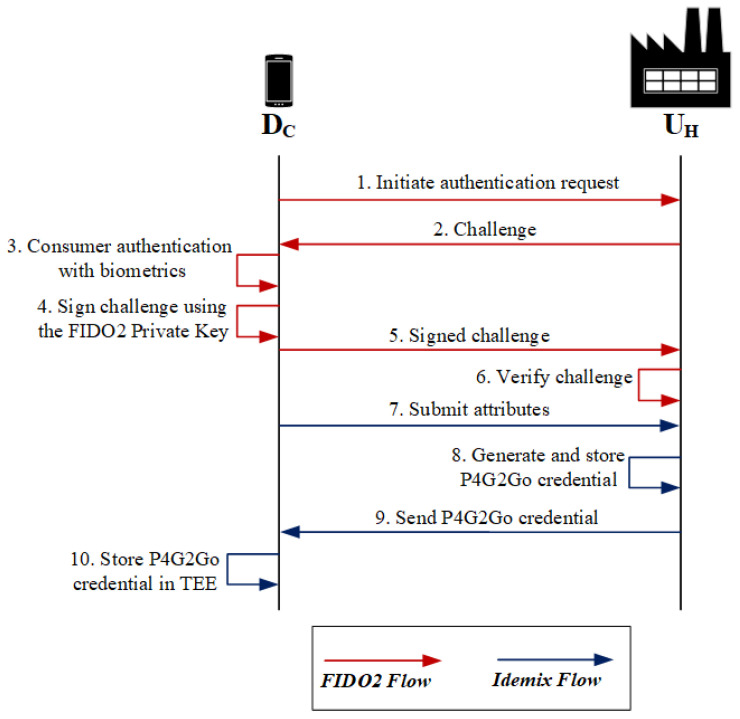
P4G2Go credential issuance.

For the credential issuance, we assume that the *Consumer* has already performed a FIDO2 registration with the *U_H_*. We also assume that the *Consumer* has generated a pseudonym *N_H_* using the Idemix master secret stored in the TEE of the *D_C_*. This pseudonym is permanent and registered in the *U_H_* (note that such pseudonyms are called domain pseudonyms in Idemix terminology). At the beginning of the credential issuance procedure, the *Consumer* performs a FIDO2 authentication as shown in Figure 3 (steps 1–6). After the *Consumer* is successfully authenticated, the *U_H_* verifies that the *Consumer* does not have unsettled debts with the *U_H_* and they are eligible to use the roaming service. If this verification is successful, the *U_H_* signs the attributes and issues an anonymous credential for this specific *Consumer* (steps 7–9). We assume that the latter stores the credentials inside a TEE in the *D_C_* (step 10). An example of a P4G2Go credential that includes a set of attributes is shown in Table 2.

The most important attribute of the P4G2Go credential is PK_U_H__(*N_H_*), which is the encryption of the *Consumer’s* pseudonym *N_H_* using the public key of the *U_H_*. Note that the attribute PK_U_H__(*N_H_*) will be used by the *U_H_* for billing the *Consumer*, as we analyze in Section 5.3.

This attribute is a verifiable encryption of the pseudonym *N_H_* as discussed previously. Other attributes in the credential are the *Consumer* details, which include various identity attributes for the *Consumer* (e.g., age), the type of *Consumer* (e.g., whether the user represents a corporate company and is eligible for a special offer), type of appliances that will be used (e.g., whether the *Consumer* can charge their electric vehicle), special offers and discounts, etc.

### 5.2. Credential Verification and Privacy-Preserving Data Aggragation

**Figure 4:**
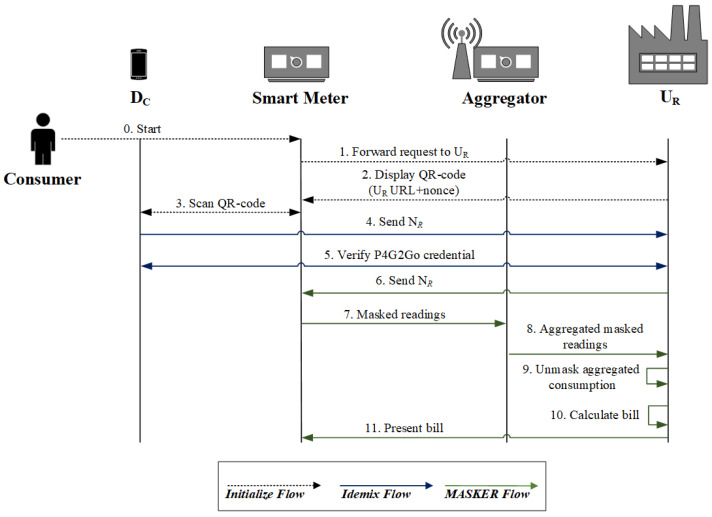
P4G2Go credential verification and energy consumption.

When the *Consumer* rents a property and wants to be charged for the energy consumption, they must provide their P4G2Go credential to the *U_R_* for validation as shown in Figure 4. To initiate the procedure, the *Consumer* interacts with the display screen of the smart meter (step 0). The latter forwards the request to the *U_R_*, which generates and sends a QR-code to the particular smart meter, which is presented on its display screen. The QR-code contains the *U_R_* URL along with a nonce value (steps 1–2). On QR-code scanning, the P4G2Go application in the *D_C_* prompts for fingerprint (or another biometric modality) authentication to unlock the P4G2Go credential stored on the TEE of the *D_C_*. On successful authentication of the *Consumer*, the *D_C_* generates on the fly a pseudonym *N_R_* using the Idemix master secret. The *Consumer* can generate as many pseudonyms as they want, which cannot be linked by the *U_R_* or any other entity. The *D_C_* sends *N_R_* along with the nonce value (of the QR-code) to the *U_R_* (steps 3–4). The latter can identify from the nonce value the smart meter used by the specific *Consumer*. At this point, the Idemix Proving Protocol is initiated between the *U_R_* (which is the verifier) and the *D_C_* (which is the prover). The proving protocol requires the *D_C_* and the *U_R_* to agree on which attribute will be revealed and which attributes will be revealed partially (for instance, the *D_C_* can prove that an attribute value is larger or smaller than a specified constant, but the real value will remain hidden from the *U_R_*). Based on the attributes of the *Consumer*, the *U_R_* checks that the specific *Consumer* conforms to the policies of the *U_R_* regarding the use of G2Go (e.g., the *Consumer* is over 18). Moreover, during the verification process, the *Consumer* proves that the provided pseudonym *N_R_* and the *N_H_* (which is encrypted in the P4G2Go credential—see Table 2) is generated by the same master secret (step 5). After successful credential verification, the *U_R_* forwards to the corresponding smart meter, the *Consumer*’s pseudonym *N_R_* informing that the *Consumer* is a valid customer and is eligible to consume energy at the rented property (step 6). From this point, the energy consumption will be charged to the *Consumer* under the pseudonym *N_R_*. Consumption data are obfuscated thanks to the MASKER protocol which guarantees an anonymous aggregation of the smart meter readings. In particular, the smart meter masks the energy consumption readings with the addition of randomly generated values before conveying them to its corresponding aggregator (step 7). The latter aggregates the masked consumption readings for the corresponding smart meter, and periodically sends them to the *U_R_* (step 8). Finally, the latter unmasks the aggregated consumption data and calculates the electricity bill for the *Consumer* which can be presented on the smart meter display screen (steps 9–11).

### 5.3. Billing and Payment

The identity of the *Consumer* must be revealed to the *U_H_* in order to charge them for their energy consumption. To this end, the *U_R_* sends to the *U_H_* the electricity bill along with PK_U_H__(*N_H_*), which was included in the P4G2Go credential of the *Consumer*. Upon receiving this information, the *U_H_* decrypts the permanent pseudonym *N_H_* using its private key and matches it with the corresponding *Consumer* identity. After successfully retrieving the *Consumer’s* true identity, the *U_H_* can charge the *Consumer* according to the energy consumption bill received from *U_R_*. The latter will never learn the real identity of the *Consumer*.

## 6. Performance Evaluation

In this section, we analyze the performance of the core components of P4G2Go to investigate the feasibility and efficiency of the proposed scheme. We focus on the execution time of (i) issuing an Idemix credential; (ii) verifying an Idemix credential; (iii) authentication through the FIDO2; (iv) registration via the FIDO2; (v) MASKER execution time in smart meters and (vi) MASKER execution time in aggregators. For our proof-of-concept implementation, the *U_H_* and *U_R_* are implemented on a desktop PC equipped with an Intel Core i5–4590 CPU at 3.30 GHz, 8 GB RAM. The *D_C_* is a Xiaomi Redmi Note 5 Qualcomm Snapdragon 6258953, Octa-core, 2000 MHz, ARM Cortex-A53, 64-bit with Android 9. The smart meters and aggregators bearing the responsibility to execute the MASKER are implemented on a Raspberry Pi v1 with a 700 MHz single-core CPU and 512 MB RAM. The P4G2Go testbed is summarized in Table 3.

For the P4G2Go prototype, we developed and used our own implementation of Idemix in Python language, along with the open-source implementation of FIDO2 protocol provided by StrongKey [49], and our previous implementation of the MASKER protocol [50]. To evaluate MASKER, real world consumption values are required. To this end, we utilized publicly available datasets of energy consumption taken from the European Network of Transmission System Operators for Electricity (ENTSO-E) [51]. 

To assess the performance of P4G2Go, we calculated the average execution time of each process individually: (i) issuing a P4G2Go credential; (ii) verifying a P4G2Go credential; (iii) FIDO2 authentication; (iv) MASKER execution in smart meters and (vi) MASKER execution in aggregators. To calculate the average duration of each process we executed it 10 times. The results are as follows (see also Table 4):

*Issuing a P4G2Go credential*: We calculate the performance of this process by generating an Idemix anonymous credential, containing two attributes to represent the *Consumer*. The time that is required to issue an Idemix credential fluctuates from 0.9 s to 1.5 s, with an average time of 1.2 s.

*Verifying a P4G2Go credential*: We calculate the performance of this process by verifying an Idemix anonymous credential that contains two attributes to represent the *Consumer*. The time that is required to verify an Idemix credential varies from 1.4 s to 1.9 s, with an average time of 1.65 s.

*FIDO2 Authentication*: We calculate the performance of this process by authenticating the *Consumer* on the FIDO2 Authentication Server of the *U_H_*, using their fingerprint. The time that is required authenticate a *Consumer* through the FIDO2 is 3.08 s.

*MASKER execution on smart meter*: We measure the time that it takes for a smart meter to compute the masked readings and send them to its corresponding aggregator. The time that is required to complete this procedure is 0.05 s.

*MASKER execution on aggregator*: We measure the time that it takes for an aggregator to accumulate the received masked readings by the corresponding smart meter. The time that is required to complete this procedure is 0.17 s.

Overall, from the numerical results we can deduce that the overhead of the P4G2Go functions is not substantial and can be executed by the smart grid entities participating in the scheme. The most time-consuming operation is the FIDO2 authentication, which takes on average 3.08 s to execute mainly due to the fingerprint authentication that requires user intervention, which causes delays in the overall authentication process.

Moreover, we measured the average CPU utilization and memory consumption of the utilized protocols (i.e., MASKER, FIDO2 and Idemix) as shown in Table 5. Regarding MASKER, we observed that the CPU utilization in the smart meter is 5.1%, while for the aggregator is 6.6%; the memory consumption is 4.8 MB and 5 MB in the smart meter and aggregator respectively. The results for *U_R_* are negligible and are not shown. Therefore, we can observe that MASKER is indeed lightweight and efficient even for devices with limited resources. For the FIDO2 protocol, the CPU utilization in the *D_C_* is 10% and 27% for registration and authentication, respectively. On the other hand, the memory consumption was around 60 MB for both registration and authentication. Additionally, for the *U_H_* that undertakes the responsibility to execute the FIDO2 processes for the server side, the CPU utilization is 5% and 2.6% for the authentication and registration respectively; the memory consumption is accordingly 1148 MB and 1158 MB. The reason behind the higher memory consumption is due to the full-fledged FIDO2 server (i.e., StrongKey server) that was utilized in the experiments. Additionally, the CPU utilization for issuing an Idemix credential containing two attributes is 17% and 26% for *D_C_* and *U_H_* respectively, and for verifying the same credential the CPU utilization reaches 28% in *U_H_*. The memory consumption during the issuance process is at 4.61 MB and 4.76 MB for *D_C_* and *U_R_* respectively. Moreover, the verification process demands 4.78 MB of memory in *U_R_*; the CPU utilization of the verification process taking place in *D_C_* is not shown, since it is negligible (the *D_C_* does not participate in the Idemix verification). Overall, based on our experiments, we argue that the individual components of P4G2Go do not deplete the resources of the participating entities, even for constrained devices such as smart meters.

**Figure 5:**
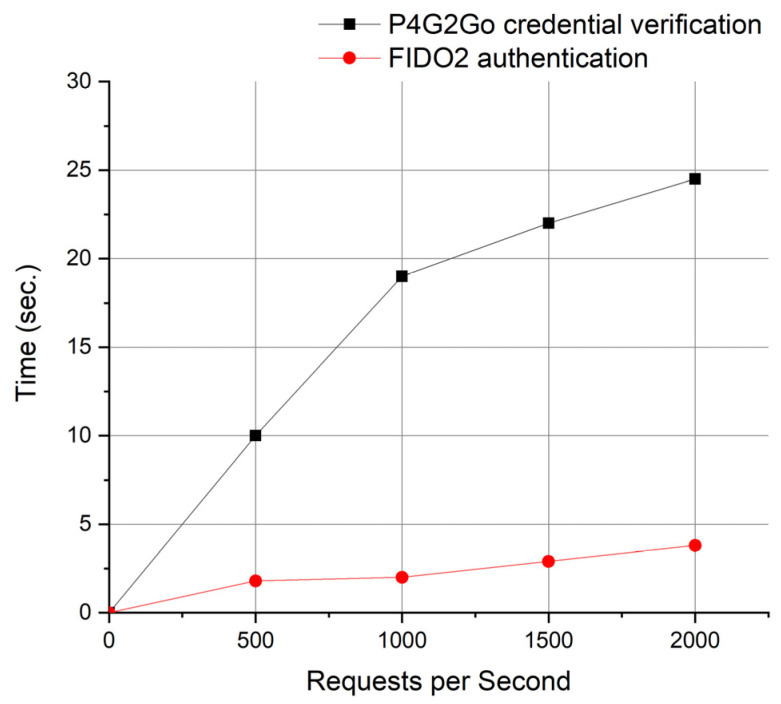
Average Response Time of P4G2Go credential verification vs. FIDO2 authentication.

Finally, we assess the performance of the proposed P4G2Go credential verification against the performance of the vanilla FIDO2 authentication. The aim of this experiment is to assess the overheads imposed by the use of anonymous credentials instead of a non-anonymous authentication solution such as FIDO2. The experiments were carried out by sending multiple authentication requests per second (from 1 to 2000 requests). The aim here was to measure the response time (average) to complete the authentication process. We used a desktop PC to emulate the *D_C_* and we simulated concurrent authentication requests using different software threads. To conduct the experiments, we utilized the Locust tool [52], a Python load testing tool, to generate valid traffic load towards our server that provided us with the average response time for each request of the processes under examination. The results were obtained for both Idemix verification and FIDO2 server authentication as shown in Figure 5. The juxtaposition of the two graphs suggests that the Idemix verification presents a non-negligible overhead compared to the vanilla FIDO2 authentication. This is a sheer showcase of usability-security tradeoff as FIDO2 authentication does not provide anonymity. However, we observe that the P4G2Go scheme can efficiently operate a significant number of parallel credential verification requests (up to 500 requests per second). The impact on the average response time is increased critically when going above 500 authentication requests per second, suggesting that the server requires more resources (scale up) or replication (scale out) to handle efficiently the workload. Note that concurrent authentication requests higher than 500 can be considered unrealistic for our scenario.

## 7. Security and Privacy Analysis

In this section, we perform the security and privacy analysis of our scheme. We argue that P4G2Go meets all privacy and security requirements presented in Section 3.3, except for physical protection (*S6* requirement as presented in Section 3.3) as hardware security can be considered out of scope of this work. P4G2Go delivers a privacy-preserving solution that can assure that the *U_R_* will not be able to identify the identity of the specific roaming *Consumer*, who is away from their home and not being served by the *U_H_* (*P1-Identity privacy*) they have a contract with. This observation can be further extrapolated in two different cases. First, the *U_R_* cannot link the *Consumer* even if the same roaming *Consumer* is visiting the same property multiple times with the same P4G2Go credential (*P3-Unlikability*). This is a direct result of the Idemix, which allows multi-showing of credentials (in contrast to another popular anonymous credential called U-Prove [53] which breaks the unlinkability property if the same credential is shown twice). Moreover, using Idemix anonymous credentials we achieve to reveal only specific attributes of the *Consumer* (*P4-Minimum data disclosure*). The second case is that our solution guarantees that no colluding parties (i.e., two or more *U_R_*) can join efforts to enhance their linking capabilities. In other words, any attempt by two or more *U_R_* to collaborate and exchange information for tracking a specific *Consumer*’s movement activities and disclose their private information will fail (*P2-Location privacy*). Again, this is a direct outcome of Idemix as well as the use of different pseudonyms for each different *U_R_*. Finally, the use of credentials and specific attributes allows only legitimate consumers to use the service made available through G2Go (*S4-Authorization and access control*).

Another important aspect of the proposed framework is related to the fact that it achieves balance between anonymity and accountability. This feature is inherited by Idemix since the latter is capable of handling potential abuses of anonymity. Accountability in smart grids is of utmost importance. This happens due to the criticality of the underlying operations of the smart grids and a potential malign *Consumer* who may cause power disruptions in extreme cases. Accountability of the P4G2Go credential can be easily achieved by the *U_H_* as it is the entity that can identify all consumers when the *U_R_* sends the PK_U_H__(*N_H_*) value for billing purposes (*S5-Accountability*).

On the other hand, it should be noted that the *U_R_* learns the *U_H_* of the *Consumer* and their total energy consumption. The *U_H_* can be considered a private information, but in order to charge the *Consumer*, the specific *U_H_* should be revealed to the *U_R_*. One evident solution to this problem is the addition of a third party, which will act as a payment broker between the *U_H_* and the *U_R_*. However, we have opted for a solution which is free of third parties, since it introduces additional layers of trust, single point of failure and deployment issues.

A usual approach proposed in the literature for storing Idemix credentials is smart cards [54], an unwieldy solution that undermines the overall user experience. P4G2Go resolves this issue by using mobile devices which users habitually carry. The positive effects of utilizing mobile devices are not limited only to usability improvements, but also to security fortifications. The omnipresence of TEE in mobile devices [55] guarantees that sensitive information is security stored. In particular, the Idemix anonymous credentials, and more importantly the Idemix master secret, are stored securely in the TEE of the *D_C_*. As the Idemix master secret is the equivalent of a private key, adversaries may target it through malicious software. If the master secret is revealed, then the security of Idemix may be compromised. However, the use of TEE hinders malware from executing arbitrary code and accessing the stored secret since TEE has the highest privileges in the OS. As a result, malware must also find an exploit to break TEE in order to read private information stored in the secure world [44]. On the other hand, the Idemix master secret being the equivalent of a private key, can be considered as a solution for non-repudiation (*S3-Non-repudiation*). The use of a mobile device as a credential wallet has another positive side-effect; it allows P4G2Go to take advantage of FIDO2 to promote passwordless authentication using strong authentication modalities, such as biometrics. Coupling FIDO2 and Idemix seems to be a promising approach allowing *Consumers* to issue Idemix credentials and store them in the TEE of a mobile device. 

As *U_R_* and aggregators are considered honest but curious (see Section 3.2), one source of concern is that patterns of consumption may reveal more information regarding a customer and their movements. However, this is not possible in P4G2Go, since the exact goal of MASKER is to prevent such privacy breach attempts. In particular, the obfuscation of consumption values by adding randomly generated values (called masks) entails data confidentiality and integrity against honest-but-curious entities which is the most widely used model in the related literature (*S1-Data confidentiality* and *S2-Data integrity*). Note that if an aggregator forwards the received masked values without aggregating them, then it does not follow the model of honest-but-curious entities, because the proper execution of the involved protocol is violated. Even if the aggregator is not trustful and misbehaves, the latter cannot obtain the readings values. Important to note also that it has been proven that the protocol does not leak information that could lead to data eavesdropping [11]. In other words, an adversary cannot deduce readings just by observing the transmitted data. MASKER preserves also the accuracy of the consumption values, because the utility can reverse the process of masking and recover the aggregated measurements exactly (*P5- Privacy-preserving data aggregation*). Moreover, MASKER utilizes a TEE not only for secure storage of keys, but also for executing sensitive operations from a security point of view including generation of masks, addition of masked values with readings, etc.). On the contrary, a TPM would not be able to perform arbitrary operations like MASKER requires, since a TPM is able to execute only a limited set of standard cryptographic operations. In this way, malicious software injected at the level of smart devices cannot penetrate and obtain sensitive cryptographic information. It is worth mentioning that the feasibility of implementing MASKER as a trusted application has been analyzed in [56]. In contrast to software attacks, hardware attacks are possible to smart meters in P4G2Go, since generally TEEs are not designed to withstand hardware attacks. There is a movement from the European Union [57] to provide available techniques for enhancing cybersecurity and privacy in Smart Metering Systems. Besides, ENISA [58] has mentioned the importance of smart grid hardware security. The literature includes works that propose the use of a TPM [59] to enhance the hardware security of smart meters, while smart cards [26] and PUFs [20] have been proposed for V2G networks.

Finally, a remark indirectly related to the security characteristics of the proposed scheme is that instead of designing new protocols from scratch, we have opted for a solution that integrates long established protocols and technologies. More specifically, P4G2Go is composed of solutions that have been extensively analyzed, and up to now, there are no imminent threats that can break their security and privacy properties. This makes P4G2Go not only more secure but also easier to implement and deploy.

## 8. Conclusions

The research outcomes of this paper can be extended in many ways as a future work. First, this work introduced for the first time the G2Go concept, which can be further analyzed from a functionality and architectural point of view. Use cases and scenarios that showcase the beneficial aspects of the proposed G2Go can be analyzed in-depth to underscore the novelty and its relevance to digital nomads. Another future direction could be towards decentralizing the architecture of the P4G2Go. This can be achieved using the notion of decentralized identifiers (DIDs). According to the W3C [60], a decentralized identifier, or DID, is “*a globally unique identifier that does not require a centralized registration authority because it is registered with distributed ledger technology or other form of decentralized network*”. Therefore, blockchain technology can be utilized to achieve a decentralization of the P4G2Go, in order to avoid placing trust in specific entities. Moreover, except for Idemix, other anonymous credential solutions can be utilized such as Anoncreds 2.0 that use the lightweight BSS+ signature [61] instead of the CL signature of Idemix. Finally, anonymous payments can be also considered by utilizing cryptocurrencies.

This paper is the first to introduce G2Go, which is a new concept realized over the smart grid, designed for traveling energy consumers who occasionally stay in places other than their home, within or outside the borders of their country of permanent residence. As the G2Go stands between the fixed-location smart grids and the mobility-enabled V2G networks, it inherits the security and privacy considerations of both. Based on this observation, this paper proposed, designed and implemented P4G2Go, a novel privacy-preserving scheme that provides strong security and privacy assertions for roaming consumers against honest but curious utilities. P4G2Go is composed of cryptographic solutions and protocols that have been analyzed, and up to now, no security flaws have been identified that could undermine their security and privacy assurances. The crux of P4G2Go is the Idemix cryptographic protocol suite, which allows roaming consumers to hide their real identity from roaming Utilities and also provide unlinkability between different showings of the consumer credentials. In P4G2Go, smart meter readings are hidden by simply adding masking values to preserve confidentiality in a lightweight manner. This is achieved by the MASKER protocol which is another critical component of our solution. We have evaluated the performance of P4G2Go and showed that it can cope with high demand as it scales well without affecting the average response time. Finally, we performed a security and privacy analysis of P4G2Go to prove that it fulfils the requirements of G2Go.

As the number traveling consumers is expected to increase over time, new privacy and security challenges will emerge. Digital nomads are becoming the standard way of remote working and may soon become the prime target of adversaries that seek to find their way to access corporate sensitive information. We hope that the research outcomes of this work become a precursor for designing privacy-preserving schemes for the newly introduced G2Go scenario.

## Figures and Tables

**Table 1:** Main entities participating in G2Go.

Entity	Description
*Consumer*	A roaming energy consumer.
*U_H_*	Is under contract to supply the *Consumer* with energy. Issues P4G2Go credentials to its customers.
*U_R_*	Is under contract to supply the rented property with energy. Verifies roaming consumers’ P4G2Go credentials.
*D_C_*	*Consumer*’s mobile device.
Smart meter	Is bound with a property and measures the occupants’ energy consumption. Conveys the consumption readings to its corresponding aggregator.
Aggregator	Aggregates the consumption readings received by smart meters. Sends valid and accurate energy consumption data to its corresponding Utility.

**Table 2:** P4G2Go credential attributes.

Attributes	Description
PK_U_H__	The public key of *U_H_*.
PK_U_H__(*N_H_*)	The *N_H_* encrypted with the public key of the *U_H_*.
Consumer details	Various requirements depending on access control policies (e.g., the *Consumer* must be over 18).
Type of consumer	Individual, corporate.
Type of appliances	Need for high energy consumption equipment, charging electric vehicles, etc.
Discounts	Special offers for the *Consumer*.
*U_H_*	The *U_H_* of the *Consumer*.
Lifetime	The expiration date of the credential.

**Table 3:** P4G2Go testbed parameters.

Entity	Setup
*U_H_*, *U_R_*	-Intel Core i5-4590 CPU at 3.30 GHz, 8 GB RAM (Download: 90.44 Mbps; Upload: 93.32 Mbps)
*D_C_*	-Xiaomi Redmi Note 5, Octa-core, 2000 MHz, ARM Cortex-A53, 64-bit, Android 9 (Download: 12.9 Mbps; Upload: 1.1 Mbps)
Smart meter, Aggregator	-Raspberry Pi v1 (a single-core 700 MHz CPU, and 512 MB–400 MHz RAM) (Download: 6.9 Mbps; Upload: 0.5 Mbps)

**Table 4:** Average duration of P4G2Go processes.

P4G2Go Processes	Average Duration (in Seconds)
Issuing a P4G2Go credential	1.2
Verifying a P4G2Go credential	1.65
FIDO2 Authentication	3.08
MASKER execution on smart meter	0.05
MASKER execution on aggregator	0.17

**Table 5:** P4G2Go overhead.

Entity	Technology	Process	CPU Utilization	Memory Consumption
*U_H_*	FIDO2	Authentication	2.6%	1158 MB
		Registration	5%	1148 MB
	Idemix	Issuance	26%	4.76 MB
*U_R_*	Idemix	Verification	28%	4.78 MB
*D_C_*	FIDO2	Authentication	27%	61.7 MB
		Registration	10%	60 MB
	Idemix	Issuance	17%	4.61 MB
Smart meter	MASKER	Masking readings	5.1%	4.8 MB
Aggregator	MASKER	Aggregating masked readings	6.6%	5 MB

## Data Availability

Not applicable.
